# Direct-Acting Antiviral Treatment in Albanian Patients With Chronic Hepatitis C and Advanced Liver Fibrosis

**DOI:** 10.7759/cureus.32646

**Published:** 2022-12-17

**Authors:** Liri Cuko, Sonila Bele, Adriana Babameto, Irgen Tafaj, Arlinda Hysenj, Eva Shagla, Agron Dogjani

**Affiliations:** 1 Department of Gastro-Hepatology, University Hospital Center “Mother Teresa”, Tirana, ALB; 2 Department of Clinical Semiology and Imagery, University of Medicine, Tirana, Tirana, ALB; 3 Department of Gastro-hepatology, University Hospital Center “Mother Teresa”, Tirana, ALB; 4 Department of General Surgery, University of Medicine, Tirana, Tirana, ALB

**Keywords:** liver cirrhosis, genotype, elastography, antiviral, chronic hepatitis c

## Abstract

Background

Treating chronic hepatitis C (CHC) with direct-acting antiviral (DAA) is very effective at clearing the infection. In Albania treatment with DAA is limited to patients with liver stiffness F3-F4, and with other co-infections. The objective of this study was to evaluate the efficacy of DAA in Albanian patients with genotypes 1-5, who mostly suffer from advanced liver fibrosis.

Material and Methods

This is a retrospective study carried out at the University Hospital Center “Mother Teresa”, Tirana, during 2014-2019, including treatment-naïve and treatment-experienced patients with genotypes 1-5. All patients were evaluated with elastography and most of them were F3-F4. The primary endpoint involved the patients achieving SVR-12, or undetectable hepatitis C virus/ribonucleic acid (HCV RNA) 12 weeks after the end of treatment. In patients without a genotype, we have used a pangenotypic regimen.

Results

This study included 207 patients with a mean age of 48.9 ± 13.1 years, 56% male and 44% female; 152 (73%) were genotype 1, 24 were (11.5%) genotype 2, nine were (4.3%) genotype 3, 14 were (6.7%) genotype 4, one was (0.4%) genotype 5, and seven (3.8%) unassigned genotypes. The sustained virologic response (SVR) percentage according to genotype is discussed in the article. The overall SVR score of all the patients in our study was >93%. According to elastography, 127 (66%) were F3-F4, and 80 (38.6%) were F1-F2.

Conclusion

Treatment with DAA proved to be very effective in our patients; most of them had advanced liver fibrosis as well as compensated or decompensated liver cirrhosis. The overall SVR score of the patients in our study was >93%. Our country needs to treat all patients with chronic hepatitis C without limitations to attain the WHO objective of eradicating this disease by 2030.

## Introduction

Hepatitis C virus (HCV) infection remains a major problem worldwide due to its high prevalence; about 71 million people globally are infected with this virus. By the time of infection, about 70% develop chronic HCV infection, and the risk for cirrhosis ranges from 15-30% within 20 years [1,2]. Numerous studies have shown that direct-acting antiviral (DAA) classes for the treatment of hepatitis C virus infection lead to the clearance of the virus from the body which shows that it is the drug of choice, effective, and works within a short period of 12 weeks to 24 weeks in advanced cirrhosis. As a result of this treatment, patients have fewer side effects, significant improvement, and shorter treatment periods. New antivirals with genotypic and pangenotypic action led to the eradication of C virus infection in over 95% of cases regardless of the degree of hepatic fibrosis, but access to diagnosis and treatment of cases remains limited [3-6]. The WHO at a meeting of its assembly in 2016 decided on a strategy for the elimination of C virus infection by 2030. This requires elimination programs to achieve the appropriate WHO target but also depends on national policies depending on the income of different countries [2]. Currently in our country since 2014 we have treated patients with chronic hepatitis C infection with advanced fibrous grade F3, F4, decompensated cirrhosis, and cases with F1, and F2 but with other comorbidities. However, the results of all treated patients regardless of the degree of fibrosis were good where >93% of patients were HCV RNA negative after DAA treatment (SVR12). The choice of medication and duration of treatment depends on the genotype of the C virus, the degree of liver damage, other medications used, and whether there are other comorbidities [7,8].

The prevalence of HCV infection in Albania according to data from the Institute of Public Health varies from 0.9-1%. The prevalence according to genotypes varies widely 73% for the 1b genotype, and 25% for other genotypes like 2, 3, 4, and 5. Although we have treated both drug regimens with genotypic and pangenotypic action, the possibility of treating all people infected with the hepatitis C virus remains uncertain due to our country's policies. For the treatment of patients, we have relied on the guidelines of the European Society of Hepatology (EASL) [8]. The treatment of these patients was performed in the only tertiary center and the purpose of this study is to evaluate the effectiveness of DAA in previously untreated patients and those treated with pegylated interferon (PEG-INF) with or without ribavirin according to the respective genotypes where the largest number of patients had genotype 1b.

## Materials and methods

This is a retrospective descriptive study, which included 207 cases (treatment-naive and treatment-experienced) followed in the gastro-hepatology department of the University Hospital Center “Mother Teresa” during the period 2014-2019, which is the only tertiary center dealing with the treatment of hepatitis C in Albania. The treatment regimens used were initially with genotypic action and later with pangenotypic action in 45 cases. We have evaluated the treatment outcomes in all cases treated according to genotypes 1-5, where most of the patients were with advanced fibrosis F3-F4. Statistical tests were used to assess treatment efficacy, side effects, and virological response (SVR). Elastography expressed in kPa was used to assess the degree of hepatic fibrosis, where F4 was considered cirrhosis. All patients were treated for 12 weeks with DAA with genotypic and pangenotypic action, while for treating decompensated cirrhosis ribavirin was used according to the patient's hemoglobin status. Ribavirin was initially used at a dose of 1200 mg for those that > 75 kg, and 1000 mg if they were <75 kg. Patients were evaluated at four, eight, and 12 weeks of treatment, clinically and according to laboratory analysis. In cases where the Hb was below 8 g/dl, ribavirin was discontinued and resumed when anemia improved. Initially, before starting treatment, the testing for the following was conducted: blood cell (WBC), hemoglobin, albumin, platelets, aspartate aminotransferase (AST), alanine aminotransferase (ALT), gamma-glutamyl transferase (GTP), HCV RNA titer, HCV genotyping, prothrombin time (PT), international normalized ratio (INR), glucose and imaging (abdominal ultrasonography). In patients with cirrhosis, liver function was assessed based on Child-Pugh criteria and the Model for End-Stage Liver Disease (MELD) score. The treatment was considered effective when HCV RNA was undetectable after 12 weeks of treatment, i.e., less than <15 UI/ml. Statistical analysis was evaluated using the SPSS software v.16 to analyze means, standard deviation, and percentage frequency expression (SPSS Inc., Chicago, IL). A p-value of <0.05 was considered statistically significant.

## Results

Of the 207 patients in the study, the mean age was 48.9 ± 13.1 years, with male predominance being 56% (117) and female 44% (90). According to the genotype, most were genotype 1 (152; 73%), where 1b predominated (69%), 24 (11.5%) were genotype 2, nine (4.3%) were genotype 3, 14 (6.7%) were genotype 4, seven (3.8%) were unassigned genotypes and one case was genotype 5. Based on the assessment of the degree of hepatic fibrosis, most of the patients were F3-F4, 127 (66%), and F1-F2 80 (38.6%); 97 (46.8%) cases were with cirrhosis (compensated and decompensated). The patients previously treated with PEG-INF and ribavirin were 106 (51%). Two patients with decompensated cirrhosis discontinued treatment due to side effects, as they had MELD scores > 20. The efficacy of both genotypic and pangenotypic treatment regimens was determined using SVR-12. Demographic and clinical data for all patients who started treatment are described in Table 1.

**Table 1:** Demographic characteristics for patients (n=207) ALT: alanine aminotransferase; AST: aspartate aminotransferase; F: fibrosis stage; HCV: hepatitis C virus

Variables	Mean, SD, %
Male (years)	117 (56%)
Age	48.9 ± 13.1
Cirrhosis	97 (46.8%)
ALT (U/L)	84.013 ± 66.7639
AST (U/L)	72.185 ± 55.8265
Bilirubin (mg/dl)	1.04572± 0.778302
F1-F2	80 (38.6%)
F3	29 (14%)
F4	98 (47.3%)
Platelets (mm^3^)	169468 ± 83.5506
HCV genotype	
1 a	23 (11%)
1b	129 (62.3%)
2	24 (11.5%)
3	9 (4.3%)
4	14 (6.7%)
5	1 (0.5%)
Genotypic treatment	152 (73%)
Pangenotypic treatment	55 (26.5%)
Treatment-naive	101 (48.7%)
Treatment-experienced	106 (51%)

The mean age in the cirrhotic group was 51.5 years of age and in the non-cirrhotic group 49.8 years of age. The mean of HCV RNA measured by PCR in non-cirrhotic patients was 2965000 UI/ml and in cirrhotic patients was 1210000 UI/ml, higher than the first group. From the data evaluated in both cirrhotic and non-cirrhotic groups the platelet count (p <0.001) hepatic fibrosis rate (p <0.001), age (p=0.036), and HCV RNA (p=0.038) were statistically significant (Table 2). 

**Table 2:** Characteristics of cirrhotic and non-cirrhotic group SVR: sustained virologic response; HCV: hepatitis C virus; RNA: ribonucleic acid

Variables	Overall (207)	Non-cirrhosis (n=110)	Cirrhosis (n=97)	p-value
Age, median	48.9 ± 13.1	49.8	51.5	0.036
SVR-12 (%)	205 (99%)	110 (100%)	95 (97.9%)	0.069
Platelet count (mm3)	169468 ± 83.5506	199000±64 470	126000±55430	< 0.001
HCV RNA	2120000±3210000	2965000 ± 3210000	1210000±245670	0.038
Transient elastography(kPa)	7.8 ± 15.3	8.1±5.9	12.3 ±10.7	< 0.001

The efficacy of treatment with both genotypic and pangenotypic regimens (DAA) for chronic hepatitis C virus infection was determined by SVR-12. Of the 207 patients, 193 showed SVR-12 (93.2%), although most of them were advanced fibrosis and had been treated before with PEG-INF and ribavirin. We should also mention the fact that cirrhotic patients were treated for only 12 weeks despite the fact that some (32) were decompensated, due to the limitations we have in our country (Table 3).

**Table 3:** SVR-12 according to the stage of the disease and the treatment regimen SVR: sustained virologic response

Variables	Total patients (n)	SVR-12 (%)
Overall	207	193 (93.2%)
Non-cirrhotic group	110	107 (97.2%)
Cirrhotic group	97	85 (87.6%)
Naïve	101	98 (97%)
Experienced	106	91(85.8%)
Genotypic treatment	152	145 (95.3%)
Pangenotypic treatment	55	54(98%)

In the non-cirrhotic group, only three cases did not respond to treatment (SVR 97%). These patients were treatment-experienced; two were genotype 3, and one was with an unspecified genotype. From 97 only 85 (87.6%) in the cirrhotic group responded to the treatment even though they were treated for only 12 weeks and were decompensated cases. In terms of the treatment regimen, it was observed that in cases treated with a genotypic regimen, out of 152 patients, 145 responded to treatment (95.3%). With the pangenotypic regimen, out of 54 cases (98%), only one case did not respond to treatment as the patient had a decompensated cirrhosis MELD score > 20 and was genotype 1a. If we look at all patients treated with DAA (genotypic, pangenotypic) a score of SVR-12 was achieved in 93.2% of cases despite the fact that most of the patients had advanced fibrosis (127; 66%), were treatment-experienced (106; 51%), or had cirrhosis (97; 46.8%). Table 3 gives the summary of SVR in all patients treated with DAA.

## Discussion

**Figure 1:**
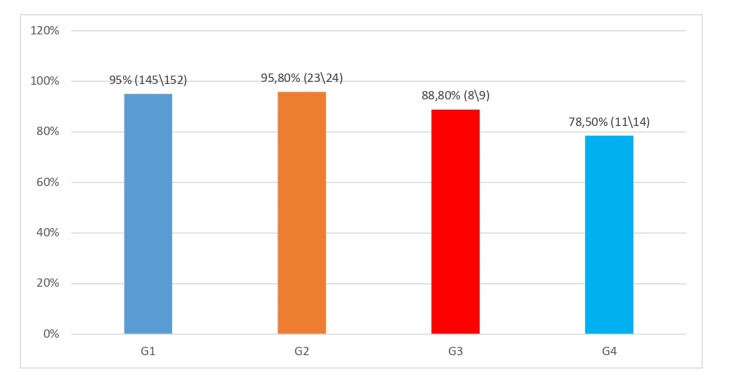
Virological response (SVR-12) in all patients according to genotypes SVR: sustained virologic response

According to the guidelines of the European Society of Hepatology (EASL), it is recommended that all individuals with chronic hepatitis C should be treated without any restrictions regardless of the degree of hepatic fibrosis except for decompensated cirrhosis when they are in advanced stages. They should have the transplant initially and be treated later [8]. It is very important to consider the risk groups for early detection of hepatitis C cases to prevent the further spread of the infection. In this study, the cause of HCV infection was not known in most cases; a good proportion of the cases were detected during blood donation (35%), with the exception of cirrhosis which had clinical and laboratory data. In the anamnesis, there were data for surgical interventions (41; 9.8%) and tattoos (35; 16.9%), and other issues. However, in a number of cases involving drug users, the patients often do not refer to themselves as users. Therefore, special programs are needed for this population, as it has been observed that 40% of drug users test positive for HCV [9]. Treatment with the pangenotypic HCV regimen includes sofosbuvir\velpatasvir and glecaprevir\pibrentasvir, which can be used without the need for genotype, thus simplifying treatment [10-12]. Genotypes should be sought in those cases where subtypes of genotypes 1-8 have been detected which are not normally present in Europe, America, Japan, and Australia. They are seen to be more prevalent in African and Asian countries [8,12], and this should be taken into account in the case of immigrants. During these years in our country, we have both treatment regimens with DAA where initially we had only genotypic therapy and last year also pangenotypic. In our study, it was seen that genotype 1 (73%) was the most predominant, followed by 1b (62%). Treatment of patients with CHC without cirrhosis and those with compensated cirrhosis (Child-Pugh class A) have shown quite satisfactory results where the SVR has been> 97% with both treatment regimens and regardless of whether they were naive or previously treated with Peg INF and ribavirin or sofosbuvir [8]. Our SVR score was> 93%, where 55 were treated with the pangenotypic regimen while the others were with the genotypic regimen. We have to keep in mind the fact that our cirrhosis has been in its advanced stages. Of 97 patients with cirrhosis, 32 (33%) had a MELD score > 20 and had been treated for only 12 weeks. This is due to the limitations of our country where incomes are relatively low. However, it was observed that in the cases treated with a pangenotypic regimen the result was 98%. Only one case failed the treatment; the patient had decompensated cirrhosis and needed transplantation. Regarding the results of treatment in patients with genotype 4, the result was 78.5% (11\14) after being treated with the genotypic regimen, in genotype 3 was 88% (8\9), but the number of cases was small, and only incomplete conclusions can be given; in genotypes 1 and 2, it was> 95% regardless of the treatment regimen (Figure 1).

This shows that we are dealing with a high SVR result regardless of the degree of fibrosis. In terms of age, there was no difference between the two groups (cirrhotic/non-cirrhotic). In this study, it was observed that patients with cirrhosis had a decrease in hemoglobin levels with ribavirin treatment, especially in decompensated cases (29\32). In these cases, the dose of ribavirin was reduced following the progress. Comparing the group of cirrhotic patients with non-cirrhotic patients, a change in platelet count was seen; a lower platelet count in cirrhotic patients indicates impaired liver function. Improving access to treatment for hepatitis C should be a priority in order to achieve WHO objectives, as well as to prevent the spread of infection [2]. However, there are still many obstacles that hinder HCV treatment, ranging from the number of infected individuals, the cost of treatment, the cost of diagnostic tests, insufficient information, and the genotype-based treatment strategy which should be simplified with the pangenotypic regimen. In conclusion, we can say that from the results of this study, individuals with CHC should be included in the treatment without any restrictions, and without the need to perform a genotype. Treatment with the pangenotypic regimen is quite successful and tolerable without any major side effects.

## Conclusions

Our study found that treatment with DAA proved to be very effective; most of our patients had advanced liver fibrosis and compensated or decompensated liver cirrhosis. The overall SVR score of all our patients was >93%. The results for genotypes 3 and 5, are not significant due to the small number of patients. In order to meet the WHO objectives, our country needs to treat all patients with chronic hepatitis C without limitations.
